# Outcomes of Patients With Unresectable Cholangiocarcinoma After Portal Vein Embolization: A Propensity Score‐Matched Analysis

**DOI:** 10.1002/jhbp.12192

**Published:** 2025-08-01

**Authors:** Ho Seung Lee, Tae Jun Song, Sung Hyun Cho, Gunn Huh, Dongwook Oh, Jae Min Lee, Jae Hoon Lee, Dae Wook Hwang, Dong‐Wan Seo

**Affiliations:** ^1^ Department of Internal Medicine Korea University Anam Hospital Seoul Korea; ^2^ Department of Gastroenterology University of Ulsan College of Medicine, Asan Medical Center Seoul Korea; ^3^ Division of Hepatobiliary and Pancreatic Surgery, Department of Surgery Asan Medical Center Seoul Korea; ^4^ Division of Gastroenterology, Department of Medicine Faculty of Medicine, Chulalongkorn University Bangkok Thailand

**Keywords:** biliary drainage, cholangiocarcinoma, portal vein embolization, propensity score matching, survival rate

## Abstract

**Background:**

This study aimed to evaluate the outcomes of patients with unresectable cholangiocarcinoma (CCA) who underwent portal vein embolization (PVE) with a focus on overall survival (OS) and the frequency of biliary drainage (BD).

**Methods:**

In this retrospective analysis, we evaluated 255 patients with unresectable CCA; 56 patients underwent PVE but ultimately remained unresectable. Propensity score matching (PSM) was used to minimize the potential confounding factors. The primary outcome was the OS, while the secondary outcome was BD frequency.

**Results:**

The PVE‐unresectable group showed lower OS than that in the non‐PVE‐unresectable group both before and after PSM (median OS: 238.5 vs. 371.0 days, *p* = 0.006; 238.5 vs. 483.5 days, *p* = 0.002, respectively). Unresectable PVE status was a predictor of worse survival both before and after PSM (hazard ratio [HR] = 2.06, *p* < 0.001 and HR = 2.46, *p* < 0.001, respectively). Chemotherapy improved survival before and after PSM (HR = 0.45, *p* < 0.001 and HR = 0.41, *p* = 0.003, respectively). The BD frequency was higher in the PVE‐unresectable group than in the non‐PVE‐unresectable group before and after PSM (0.693 vs. 0.470 procedures per month, *p* = 0.010).

**Conclusions:**

Patients with unresectable CCA who underwent PVE had worse survival outcomes and required BD. Optimizing systemic therapy and BD strategies may improve the outcomes.

**Abbreviations**
AE: adverse event
BD: biliary drainage
BMI: body mass index
CCA: cholangiocarcinoma
CCI: Charlson Comorbidity Index
CEA: carcinoembryonic antigen
FLR: future liver remnant
HR: hazard ratio
IRB: Institutional Review Board
O&C: open‐and‐close
OS: overall survival
PHLF: post‐hepatectomy liver failure
PSM: propensity score matching
PVE: portal vein embolization

## 1 Introduction

Portal vein embolization (PVE) is a preoperative technique used to stimulate the growth of the future liver remnant (FLR) by redirecting portal blood flow [1, 2]. In some cases, it is followed by hepatic vein embolization to further enhance liver hypertrophy [3]. This approach is particularly important for patients requiring major liver resection, as an inadequate FLR significantly increases the risk of post‐hepatectomy liver failure (PHLF) [4, 5]. In cholangiocarcinoma (CCA), an FLR of at least 40% is generally considered necessary to proceed safely with surgery [6, 7, 8, 9].

Patients who undergo surgery after PVE typically experience favorable surgical and oncological outcomes [10, 11]. In addition to enhancing FLR volume, PVE reduces postoperative adverse events (AEs), particularly in patients with underlying liver disease [12, 13, 14], and enables safer major hepatectomies by enhancing the liver's regeneration capacity before surgery [4, 6]. PVE is considered a safe procedure, with reported major AE rates ranging from approximately 0.32% to 1.6% [6, 7, 15]. However, in CCA, surgery remains infeasible in up to 22% of patients because of factors such as metastasis or inadequate liver regeneration following PVE [7]. A single study reported an increased risk of AEs—including liver abscess formation—in up to 31% of patients when the embolized liver lobe was not resected [16]. The long‐term consequences of retaining an embolized lobe in patients who do not undergo surgery remain poorly understood.

Despite these challenges, there has been little research examining the prognosis and subsequent management of patients who undergo PVE but ultimately remain unresectable. This study evaluated the outcomes in these patients and explored potential strategies to optimize their management.

## 2 Methods

### 2.1 Patients and Procedures

We retrospectively reviewed the CCA cohort database at the Asan Medical Center to collect data on patients initially deemed unresectable, including those who underwent PVE in preparation for surgical resection but ultimately did not undergo surgery, and those who did not receive PVE between March 2017 and December 2022. The patients included in this study were aged > 19 years and had biopsy‐confirmed CCA. Patients with a concurrent malignancy at the time of CCA diagnosis or a history of cancer within 5 years prior to CCA diagnosis were excluded from the analysis.

PVE was performed using a combination of embolic materials, including Interlock detachable coils, N‐butyl‐2‐cyanoacrylate (NBCA, Histoacryl) diluted with Lipiodol, and Gelfoam sponge. The selection and combination of materials were based on vascular anatomy and operator preference.

### 2.2 Data Collection

Data were collected from electronic medical records and included patient demographics (sex, age, body mass index [BMI], and Charlson Comorbidity Index [CCI]), laboratory test results (total bilirubin, serum albumin, carcinoembryonic antigen [CEA], and carbohydrate antigen [CA] 19–9), and clinical characteristics such as CCA type and cancer stage. Chemotherapy and radiotherapy statuses were recorded to determine whether patients received these treatments at any point during the course of the disease. Details of the PVE procedure, including the date, targeted portal vein branch, and reasons for not proceeding with the surgery, were also collected. Biliary drainage (BD) data included the BD method; date of initial BD; frequency of BD procedures at 6, 12, 24, 36, 48, and 60 months after the initial hospital visit; and the total number of BD procedures performed throughout the follow‐up period. In this study, the term “BD” collectively refers to endoscopic retrograde cholangiopancreatography, percutaneous transhepatic biliary drainage—including drainage performed for liver abscess—and endoscopic ultrasound‐guided biliary drainage. The frequency of BD was calculated by including all relevant procedures, such as stent placement, stent exchange, insertion of percutaneous biliary drainage tubes, and tube replacement. Survival data included overall survival (OS), time to death or last follow‐up, and survival duration. Ethical approval for this study (IRB number: 2023‐0167) was provided by the Institutional Review Board of Asan Medical Center, Seoul, Republic of Korea on February 6, 2025.

### 2.3 Multidisciplinary Treatment Planning and PVE Indications

All included patients were evaluated by a multidisciplinary team, and treatment decisions were made by consensus. Volumetrically, PVE was performed when the FLR was expected to be less than 40% in patients with CCA or when the surgeon or physician considered it necessary owing to a high risk of PHLF. Chemotherapy was administered according to the standard guidelines and tailored to individual patient needs. An oncology specialist performed all oncological treatments. Access to chemotherapy was comparable between the two groups. In the PVE‐unresectable group, palliative chemotherapy was initiated after PVE when the patient's clinical condition permitted. Due to variability in chemotherapy regimens, patients were categorized based on whether or not they received palliative chemotherapy.

### 2.4 Propensity Score Matching and Study Outcomes

To minimize potential confounding factors, propensity score matching (PSM) was performed using a nearest‐neighbor algorithm with a 1:1 ratio and caliper of 0.2. Matching was based on age, sex, cancer stage, CCI score, tumor type, chemotherapy status, serum albumin level, total bilirubin level, and BMI. After matching, the patients were categorized into two groups: the PVE‐unresectable group, consisting of those who underwent PVE but were ultimately deemed unresectable, and the non‐PVE‐unresectable group, comprising patients who were determined to be unresectable at the time of initial diagnosis.

The primary outcome of this study was OS, which was compared between the PVE‐unresectable and non‐PVE‐unresectable groups using the Kaplan–Meier method. OS was defined as the time from the initial hospital visit to death or the last follow‐up. Factors associated with OS were also evaluated. The secondary outcome was the frequency of the BD procedures over time in both groups. The BD incidence was calculated as the number of BD procedures per unit time, accounting for the effect of survival duration on the frequency of BD.

### 2.5 Statistical Analysis

Continuous variables were summarized as mean ± standard deviation for normally distributed data or as median with interquartile range for non‐normally distributed data. Categorical variables were presented as frequencies and percentages. Comparisons between the PVE‐unresectable and non‐PVE‐unresectable groups were conducted to assess the differences in OS. PSM was used to balance key clinical variables between the groups. Survival analysis was performed using the Kaplan–Meier method, and group differences were evaluated using the log‐rank test before and after PSM. Cox proportional hazards regression was used to identify the factors associated with OS in both the pre‐ and post‐matching cohorts. To assess BD utilization, the total number of procedures per unit time was compared between the two groups before and after PSM, using the Wilcoxon rank‐sum test. Gamma regression with a log‐link function was used to analyze the factors associated with BD frequency, as this model appropriately accounted for positively skewed, time‐adjusted BD data. Statistical significance was defined as a two‐sided *p*‐value < 0.05. All statistical analyses were conducted using the R software (version 4.4.3; R Foundation for Statistical Computing, Vienna, Austria).

## 3 Results

### 3.1 Baseline Characteristics of the Study Population

**FIGURE 1:**
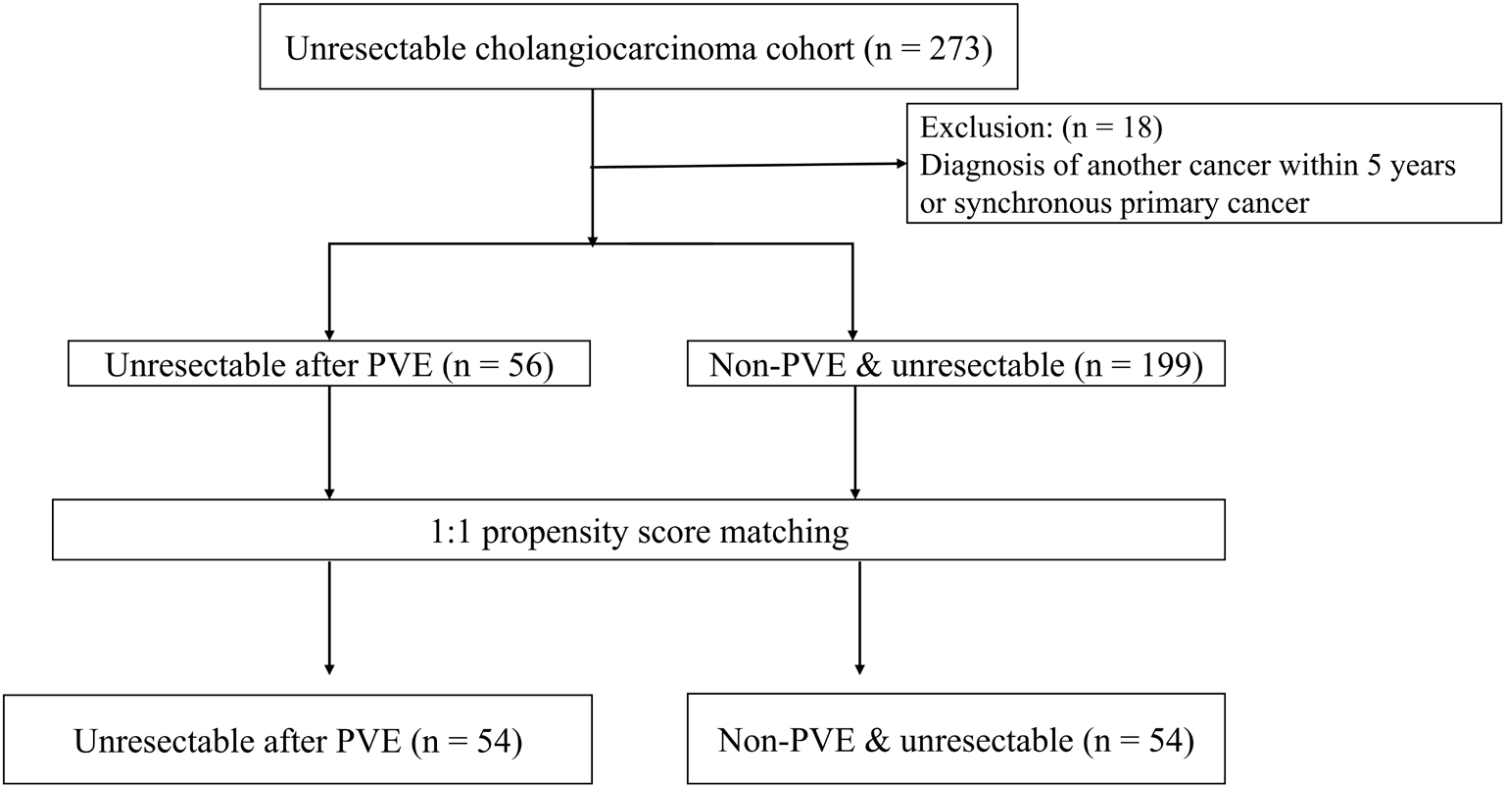
Flowchart of the study showing the selection process for eligible patients.

In total, 273 patients with unresectable CCA were identified from the database. After excluding patients with concurrent malignancy at the time of CCA diagnosis or a history of malignancy within 5 years prior to CCA diagnosis, 255 patients were included in the final analysis (non‐PVE‐unresectable, *n* = 199; PVE‐unresectable, *n* = 56). Of the 56 patients in the PVE‐unresectable group, 23 underwent an open‐and‐close (O&C) procedure. The reasons for performing O&C include peritoneal seeding, major vessel invasion, other distant metastases, local invasion (hilar or duodenal), and underlying liver cirrhosis. The remaining 33 patients did not undergo surgery because of sepsis related to cholangitis, liver abscess formation, disease progression during the preoperative period, insufficient remnant liver volume, or poor general condition. To balance the baseline characteristics between the two groups, PSM was performed, yielding a final cohort of 108 patients (non‐PVE‐unresectable, *n* = 54; PVE‐unresectable, *n* = 54) (Figure 1). The baseline characteristics of the pre‐ and post‐matching cohorts are summarized in Table 1. After matching, all measures including age, sex, cancer stage, tumor type, and biochemical markers were well balanced between the two groups, except for a persistently higher incidence of liver abscess formation in the PVE‐unresectable group.

**TABLE 1:** Baseline characteristics of non‐PVE‐unresectable and PVE‐unresectable groups before and after propensity score matching (PSM).

Before PSM					After PSM			
	Non‐PVE & unresectable (*n* = 199)	Unresectable after PVE (*n* = 56)	*p*	SMD	Non‐PVE & unresectable (*n* = 54)	Unresectable after PVE (*n* = 54)	*p*	SMD
Age (years), mean ± SD	74.47 ± 11.41	72.84 ± 8.49	0.322	0.162	73.07 (12.28)	72.48 (8.31)	0.77	0.057
Sex			0.811	0.060			1	0.038
Male:female (%)	115:84 (57.8:42.2)	34:22 (39.3:60.7)			32:22 (59.3:40.7)	33:21 (61.1:38.9)		
Stage			< 0.001	0.818			0.94	0.068
II	15 (7.5)	7 (12.5)			4 (7.4)	5 (9.3)		
III	57 (28.6)	38 (67.9)			39 (72.2)	38 (70.4)		
IV	127 (63.9)	11 (19.6)			11 (20.4)	11 (20.4)		
CCI (mean ± SD)	8.03 ± 2.21	5.95 ± 1.46	< 0.001	1.109	6.04 (1.73)	5.96 (1.48)	0.811	0.046
Cancer type			< 0.001	0.875			0.74	0.128
dCBD (%)	21 (10.6)	0 (0.0)						
IH‐CCA (%)	70 (35.2)	6 (10.7)			4 (7.4)	6 (11.1)		
PH‐CCA (%)	108 (54.3)	50 (89.3)			50 (92.6)	48 (88.9)		
Chemotherapy			0.679	0.207			0.83	0.083
*n* (%)	70 (35.2)	16 (28.6)			16 (29.6)	14 (25.9)		
*y* (%)	127 (63.8)	40 (71.4)			38 (70.4)	40 (74.1)		
BMI (mean ± SD) (kg/m^2^)	23.31 ± 3.33	24.04 ± 2.75	0.133	0.240	23.81 (3.68)	23.97 (2.77)	0.802	0.048
Total bilirubin (mean ± SD) (mg/dL)	4.54 ± 6.52	5.49 ± 7.08	0.349	0.139	4.68 (6.24)	5.32 (6.96)	0.614	0.097
Albumin (mean ± SD)	3.28 ± 0.58	3.27 ± 0.53	0.914	0.017	3.24 (0.56)	3.29 (0.52)	0.657	0.086
CA 19–9 (U/mL)			0.236				1	
≥ 37 (%)	156 (78.4)	39 (69.6)			39 (72.2)	38 (70.4)		
< 37 (%)	43 (21.6)	17 (30.4)			15 (27.8)	16 (29.6)		
CEA (ng/mL)			0.078				0.846	
≥ 2 (%)	131 (65.8)	29 (51.8)			31 (57.4)	29 (53.7)		
< 2 (%)	68 (34.2)	27 (48.2)			23 (42.6)	25 (46.3)		
Liver abscess			< 0.001				< 0.001	
*n* (%)	170 (85.4)	22 (39.3)			44 (81.5)	21 (38.9)		
*y* (%)	29 (14.6)	34 (60.7)			10 (18.5)	33 (61.1)		

*Note:* BMI, body mass index; CA 19–9, carbohydrate antigen 19–9; CCI, Charlson Comorbidity Index; CEA, carcinoembryonic antigen; dCBD, distal common bile duct; IH‐CCA, intrahepatic cholangiocarcinoma; PH‐CCA, perihilar cholangiocarcinoma; PVE, portal vein embolization; SD, standard deviation; SMD, standardized mean difference.

The indications for the first BD procedure in the PVE‐unresectable group are summarized in Table S1. The majority were performed for therapeutic reasons, such as cholangitis, liver abscess, or worsening jaundice (69.6%), while only a small proportion were related to non‐obstructive or elective purposes. The reasons for not proceeding to resection after PVE were reviewed and are summarized in Table S2.

### 3.2 Comparison of OS Between PVE‐Unresectable and Non‐PVE‐Unresectable Groups Before and After PSM

**FIGURE 2:**
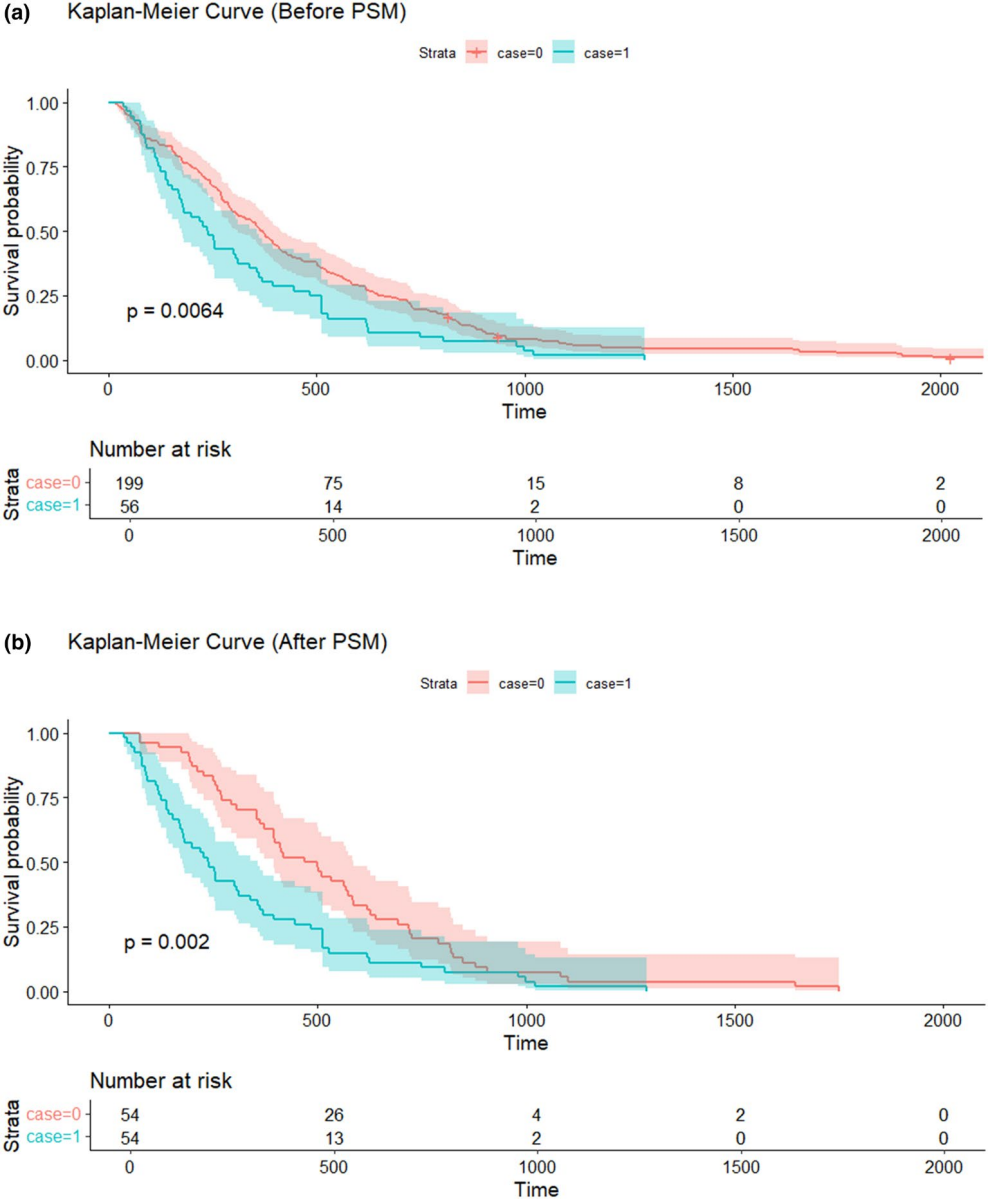
Kaplan–Meier survival curves before and after propensity score matching (PSM). (a) Kaplan–Meier survival curve before propensity score matching. (b) Kaplan–Meier survival curve after propensity score matching. This figure illustrates the differences in overall survival (OS) between the two groups before and after propensity score matching (PSM). The PVE‐unresectable group (case = 1) and the initially unresectable group (case = 0) were compared using the Kaplan–Meier method. Statistical significance was assessed using log‐rank tests.

Figure 2 illustrates the Kaplan–Meier survival curves comparing the OS between the PVE‐unresectable and non‐PVE‐unresectable groups before and after PSM. Before matching, the PVE‐unresectable group exhibited a significantly worse OS than the non‐PVE‐unresectable group (log‐rank test, *p* = 0.006) (Figure 2a). The median survival was 371.0 days (95% confidence interval [CI]: 312–415 days) in the non‐PVE‐unresectable group and 238.5 days (95% CI: 175–356 days) in the PVE‐unresectable group. After PSM, the difference in survival between the two groups remained statistically significant (log‐rank test, *p* = 0.002) (Figure 2b). The median survival time for the non‐PVE‐unresectable group was 483.5 days (95% CI: 394–585 days), whereas the PVE‐unresectable group maintained a median survival of 238.5 days (95% CI: 175–356 days).

### 3.3 Cox Model Results for OS Before and After PSM

**FIGURE 3:**
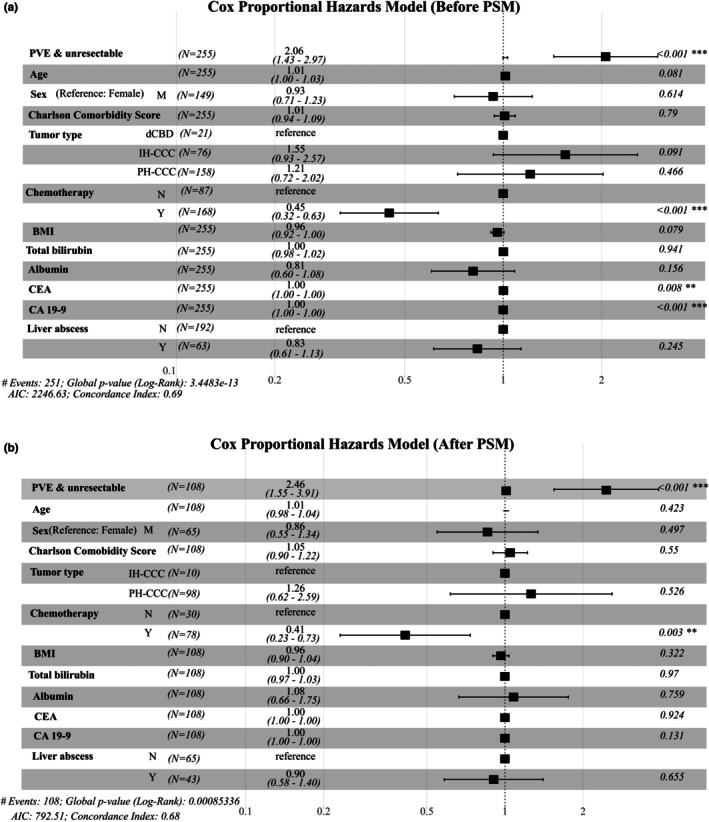
Cox proportional hazards analysis for overall survival before and after propensity score matching (PSM). (a) Cox proportional hazards analysis for overall survival before propensity score matching. (b) Cox proportional hazards analysis for overall survival after propensity score matching.

Cox proportional hazards regression analysis was conducted to identify the factors associated with OS before and after PSM (Figure 3). Before PSM, the PVE‐unresectable group had a significantly higher hazard of death than the non‐PVE‐unresectable group (hazard ratio [HR] = 2.06; 95% CI: 1.43–2.97, *p* < 0.001). Chemotherapy was associated with a significantly lower risk of death (HR = 0.45; 95% CI: 0.32–0.63, *p* < 0.001). Higher CEA and CA 19–9 levels were significantly associated with OS, although the HRs were close to 1. Other covariates, including age, sex, CCI score, tumor type, total bilirubin level, serum albumin level, and liver abscess status, were not significantly associated with OS. Following PSM, the PVE‐unresectable group remained significantly associated with an increased risk of death compared to the non‐PVE‐unresectable group (HR = 2.46; 95% CI: 1.55–3.91, *p* < 0.001). Chemotherapy remained significantly associated with a reduced risk of death (HR = 0.41; 95% CI: 0.23–0.73, *p* = 0.003). No significant associations were observed between age, sex, CCI, tumor type, BMI, total bilirubin level, serum albumin level, and liver abscesses. The C‐indices of the models before and after PSM were 0.690 and 0.684, respectively, indicating a moderate predictive performance.

### 3.4 Gamma Regression Analysis of BD Frequency

Before PSM, the median BD frequency was higher in the PVE‐unresectable group (0.693 procedure/month) than in the non‐PVE‐unresectable group (0.406 procedure/month). After PSM, the PVE‐unresectable group continued to show a higher frequency of BD (0.693 procedures/month) than did the non‐PVE‐unresectable group (0.470 procedures/month). Wilcoxon rank‐sum tests confirmed a statistically significant difference in the median BD frequency before (*W* = 3706, *p* < 0.001) and after PSM (*W* = 1038, *p* = 0.010). Gamma regression analysis was performed to identify factors associated with BD frequency before and after PSM (Table 2). Before matching, the PVE‐unresectable group showed a trend toward increased BD frequency compared with the non‐PVE‐unresectable group; however, this difference was not statistically significant (*β* = 0.25; *p* = 0.082). Chemotherapy was significantly associated with reduced BD frequency (*β* = −0.44; *p* = 0.001), whereas liver abscess formation was linked to increased BD frequency (*β* = 0.29; *p* = 0.027). Other factors, including age, sex, CCI, tumor type, BMI, total bilirubin level, serum albumin level, CEA, CA 19–9, and radiotherapy, were not significantly associated with BD frequency. After PSM, the PVE‐unresectable group had a considerably higher BD frequency than that of the non‐PVE‐unresectable group (*β* = 0.33; *p* = 0.031). Liver abscess formation remained a significant predictor of increased BD frequency (*β* = 0.34; *p* = 0.030), whereas chemotherapy (*β* = −0.42; *p* = 0.018) and higher CCI scores (*β* = −0.10; *p* = 0.041) were associated with reduced BD frequency. Additionally, perihilar CCA (PH‐CCA) was significantly associated with increased BD frequency (*β* = 0.81; *p* = 0.005).

**TABLE 2:** Gamma regression analysis of biliary drainage frequency before and after propensity score matching (PSM).

Gamma regression results before PSM				
Variable	Estimate	Standard error	*t*	*p*
Intercept	0.958	0.801	1.196	0.233
PVE and unresectable	0.250	0.143	1.746	0.082
Age	−0.008	0.006	−1.309	0.191
Sex (male)	−0.004	0.109	−0.033	0.974
CCI	0.007	0.028	0.230	0.819
IH‐CCA	−0.042	0.211	−0.198	0.843
PH‐CCA	−0.070	0.194	−0.363	0.717
Chemotherapy	−0.444	0.137	−3.256	0.001
BMI (kg/m^2^)	−0.010	0.171	−0.695	0.488
Total bilirubin (mg/dL)	0.010	0.009	1.177	0.240
CEA (U/mL)	0.0003	0.0004	0.787	0.433
CA 19–9 (ng/mL)	0.000002	0.000006	0.297	0.767
Radiotherapy	−0.058	0.173	−0.334	0.738
Albumin (g/dL)	−0.141	0.112	−1.253	0.219
Liver abscess	0.285	0.128	2.225	0.027

Gamma regression results after PSM				
Variable	Estimate	Standard error	*t*	*p*
Intercept	−0.115	0.969	−0.119	0.906
PVE and unresectable	0.330	0.150	2.196	0.031
Age	0.003	0.008	0.324	0.747
Sex (male)	0.205	0.152	1.349	0.181
CCI	−0.101	0.049	−2.077	0.041
PH‐CCA	0.812	0.282	2.877	0.005
Chemotherapy	−0.419	0.175	−2.404	0.018
BMI (kg/m^2^)	−0.006	0.217	−0.270	0.787
Total bilirubin (mg/dL)	−0.011	0.011	−0.945	0.347
CEA (U/mL)	0.001	0.0009	1.098	0.275
CA 19–9 (ng/mL)	0.00003	0.00002	1.289	0.201
Radiotherapy	0.100	0.204	0.493	0.624
Albumin (g/dL)	−0.214	0.155	−1.384	0.170
Liver abscess	0.340	0.155	2.203	0.030

*Note:* Gamma regression results before PSM: The dispersion parameter for the gamma family was estimated to be 0.594. The goodness‐of‐fit of the model was evaluated using deviance measures, with a null deviance of 152.15 for 224° of freedom and a residual deviance of 129.74 for 210° of freedom. The Akaike Information Criterion (AIC) value for model selection was 218.94. The model converged after nine Fisher scoring iterations. Gamma regression results after PSM: The dispersion parameter for the gamma family was estimated to be 0.464. The goodness‐of‐fit of the model was evaluated using deviance measures, with a null deviance of 67.039 for 104° of freedom and a residual deviance of 51.762 for 91° of freedom. The AIC value for the model selection was 111.01. The model converged after nine Fisher scoring iterations.

Abbreviations: BMI, body mass index; CA 19–9, carbohydrate antigen 19–9; CCI, Charlson Comorbidity Index; CEA, carcinoembryonic antigen; IH‐CCA, intrahepatic cholangiocarcinoma; PH‐CCA, perihilar cholangiocarcinoma; PVE, portal vein embolization.

Within the PVE‐unresectable group, there were no significant differences in OS (*p* = 0.62) or BD frequency (*p* = 0.4338) between patients who underwent O&C laparotomy and those who did not undergo laparotomy (Figure S1).

## 4 Discussion

PVE is commonly employed to reduce the risk of PHLF in patients undergoing surgery for primary liver malignancies or resectable liver metastases [5, 6, 8, 12]. While the 5‐year OS rate for patients with CCA who undergo hepatectomy after PVE has been reported to be 39% [5], there remains a notable lack of research on the prognosis and clinical outcomes of patients who undergo PVE but ultimately do not proceed to resection [13]. A previous study compared 16 unresectable patients who underwent PVE with 48 unresectable patients who did not, focusing primarily on infectious AEs such as liver abscesses [16]. However, that study did not evaluate survival outcomes or the long‐term clinical course. Addressing this gap, our study offers further insight into the clinical outcomes of patients who remain unresectable after PVE.

Survival analysis revealed that before PSM, OS was significantly lower in the PVE‐unresectable group than in the non‐PVE‐unresectable group (median OS: 238.5 days [95% CI: 175–356] vs. 371.0 days [95% CI: 312–415]). After PSM, this survival difference remained significant (median OS: 238.5 days [95% CI: 175–356] vs. 483.5 days [95% CI: 394–585]), confirming an independent association between PVE‐unresectable status and poorer prognosis. The reported median survival for patients with PH‐CCA or intrahepatic CCA receiving palliative locoregional or systemic treatment ranges from 11.7 to 14.5 months [17, 18, 19, 20], consistent with the median survival observed in the non‐PVE‐unresectable group in our study. This supports the real‐world applicability of our findings. Of note, our study showed that liver abscesses occurred in 34 patients (60.7%) in the PVE‐unresectable group, a significantly higher proportion than that in the non‐PVE‐unresectable group (*p* < 0.001). The incidence of liver abscesses in our study was higher than that reported previously (31%, 5/16) [16], possibly because of the larger sample size and exclusive focus on CCA. In contrast, an earlier study included 37.5% of patients with colorectal liver metastases, which may explain the observed discrepancies.

Several factors may account for the lower survival rate in the PVE‐unresectable group. First, these patients constitute a high‐risk population with borderline resectability, and PVE is typically performed when the feasibility of surgical resection is uncertain. In the PVE‐unresectable group, tumor progression or clinical deterioration likely rendered the patients ineligible for resection [21]. Compared with the initially unresectable group, these patients may have experienced more rapid disease progression or worsening general condition following PVE, contributing to inferior outcomes. Second, while PVE is generally considered a safe procedure, its potential AEs—such as hepatic function deterioration, portal vein thrombosis, or infection—may limit subsequent systemic therapy options and worsen prognosis [6]. Unlike the initially unresectable group, who promptly began systemic treatment, patients in the PVE‐unresectable group may have experienced delays in surgery, cancellations, and postponed systemic therapy, allowing disease progression during this vulnerable period.

Cox proportional hazards regression analysis demonstrated that, before PSM, PVE‐unresectable patients had significantly worse survival than that of non‐PVE‐unresectable patients (HR = 2.06; 95% CI: 1.43–2.97, *p* < 0.001), whereas chemotherapy was associated with improved survival (HR = 0.45; 95% CI: 0.32–0.63, *p* < 0.001). CEA and CA 19–9 were statistically significant but showed HR values close to 1, suggesting minimal clinical relevance despite statistical significance. After PSM, PVE‐unresectable status remained significantly associated with poorer OS (HR = 2.46; 95% CI: 1.55–3.91, *p* < 0.001), and chemotherapy continued to show a survival benefit (HR = 0.41; 95% CI: 0.23–0.73, *p* = 0.003). These findings underscore the poor prognosis of the PVE‐unresectable group and reaffirm the benefit of chemotherapy in advanced CCA, consistent with the findings of previous studies demonstrating the survival advantages of systemic therapy for unresectable CCA [17, 18, 19].

Our study found that the PVE‐unresectable group required more frequent BD procedures and explored the potential contributing factors. Before PSM, the median BD frequency was significantly higher in the PVE‐unresectable group than in the non‐PVE‐unresectable group (0.693 vs. 0.406 procedures/month; *p* < 0.001). This difference remained statistically significant after PSM (0.693 vs. 0.470 procedures/month; *p* = 0.010). Several mechanisms may explain the increased need for BD in this group. Right‐sided PVE without subsequent resection may lead to biliary stasis, predisposing patients to cholangitis [22], which increases the demand for BD. Additionally, hepatic changes following embolization in the non‐resected liver may contribute to recurrent biliary obstruction. Tumor progression in this borderline resectable cohort may further compromise biliary outflow, necessitating more frequent BD interventions. Several studies have suggested a potential interaction between chemotherapy and the frequency of BD in biliary tract cancers. While findings vary across studies, it is generally believed that frequent BD procedures owing to cholangitis may delay or interrupt chemotherapy administration, ultimately leading to worse prognosis [23, 24, 25]. In our gamma regression analysis, chemotherapy administration (*p* = 0.001) and liver abscess formation (*p* = 0.027) were significant factors associated with increased BD frequency, both before and after PSM (*p* = 0.018 and *p* = 0.030, respectively). These findings underscore the importance of systemic treatment in preventing bile duct obstruction. The PVE‐unresectable status showed a trend toward a higher BD frequency before PSM (*p* = 0.082), which reached statistical significance after matching (*p* = 0.031). This suggests that prophylactic BD may be selectively considered for high‐risk patients with unresectable disease following PVE to reduce adverse biliary events, although prospective validation is warranted. PH‐CCA was a significant predictor of increased BD frequency after PSM (*p* = 0.005), indicating that unadjusted analyses may have been influenced by baseline confounding. Notably, a higher CCI score was associated with a lower BD frequency after PSM (*p* = 0.041), likely reflecting clinical scenarios in which aggressive interventions were limited owing to a poor overall condition or a shift toward best supportive care.

Given the poor OS and increased BD frequency observed in the PVE‐unresectable group, these findings highlight the need for more stringent patient selection and rigorous pre‐PVE assessment to identify those most likely to benefit from the procedure. Initiation of systemic therapy prior to PVE may also be considered in selected patients with high tumor burden or suspected micro‐metastasis, as a means to allow better biological assessment and avoid unnecessary PVE in patients with rapidly progressing disease. These hypotheses warrant further investigation through prospective cohort studies or randomized controlled trials to determine whether refining patient selection criteria or incorporating pre‐PVE systemic therapy can improve clinical outcomes in this patient population.

This study has some limitations. First, its retrospective design potentially introduced a selection bias. Although PSM was employed to balance baseline characteristics, unmeasured confounders may have influenced the observed outcomes. In particular, patients selected for PVE at our institution—a high‐volume tertiary referral center—may include individuals considered unresectable at other centers, thereby reducing the clinical distinction between groups. This institutional tendency toward aggressive treatment may have affected group composition and limited external comparability. Second, the study was conducted at a single institution, which may limit its generalizability. However, the median survival of patients with initially unresectable CCA in our cohort was comparable to that reported in large‐scale studies, suggesting that our findings may be broadly applicable. Third, the study did not evaluate the effect of specific chemotherapy regimens, which could have influenced survival outcomes. Nonetheless, as no breakthrough chemotherapy regimen for CCA has been established yet, the effect of different regimens on OS may be limited. Future prospective studies with larger multicenter cohorts are warranted to validate these findings and refine the patient selection criteria for PVE.

In conclusion, this study demonstrated that patients with unresectable CCA who underwent PVE but not surgery had significantly worse survival outcomes and required BD procedures more frequently than those who were initially considered unresectable. These findings underscore the importance of careful patient selection and monitoring after PVE. Optimizing perioperative strategies, including systemic therapy and BD management, may improve the outcomes in this high‐risk population.

## Author Contributions

Conception and design: T.J.S. Data analysis and interpretation: H.S.L., S.H.C., G.H., J.M.L., J.H.L., D.W.H., D.O., D.‐W.S., and T.J.S. Drafting of the article: H.S.L. Critical revision of the manuscript for important intellectual content: T.J.S. Statistical analysis: H.S.L. and T.J.S. Supervision: T.J.S. Final approval of the article: T.J.S.

## Ethics Statement

This study was approved by the IRB of Asan Medical Center (approval number: 2023‐0167).

## Consent

The authors have nothing to report.

## Conflicts of Interest

The authors declare no conflicts of interest.

## Supporting information


**Figure S1.** Comparison of survival and biliary drainage frequency between O&C and non‐laparotomy patients in the PVE‐unresectable group.

Data S1.

Data S2.

**Table S1.** Indications for first biliary drainage after portal vein embolization (PVE) in the PVE‐unresectable group.
**Table S2**. Reasons for non‐resection after portal vein embolization.

## Data Availability

The data that support the findings of this study are available on request from the corresponding author. The data are not publicly available due to privacy or ethical restrictions.

## References

[1] O. Farges , J. Belghiti , R. Kianmanesh , et al., “Portal Vein Embolization Before Right Hepatectomy: Prospective Clinical Trial,” Annals of Surgery 237 (2003): 208–217. 12560779 10.1097/01.SLA.0000048447.16651.7B PMC1522143

[2] A. W. Hemming , A. I. Reed , R. J. Howard , et al., “Preoperative Portal Vein Embolization for Extended Hepatectomy,” Annals of Surgery 237 (2003): 686–691. 12724635 10.1097/01.SLA.0000065265.16728.C0 PMC1514515

[3] K. Araki , K. Shibuya , N. Harimoto , et al., “A Prospective Study of Sequential Hepatic Vein Embolization After Portal Vein Embolization in Patients Scheduled for Right‐Sided Major Hepatectomy: Results of Feasibility and Surgical Strategy Using Functional Liver Assessment,” Journal of Hepato‐Biliary‐Pancreatic Sciences 30 (2023): 91–101. 35737808 10.1002/jhbp.1207

[4] A. Abulkhir , P. Limongelli , A. J. Healey , et al., “Preoperative Portal Vein Embolization for Major Liver Resection: A Meta‐Analysis,” Annals of Surgery 247 (2008): 49–57. 18156923 10.1097/SLA.0b013e31815f6e5b

[5] T. Ebata , Y. Yokoyama , T. Igami , G. Sugawara , Y. Takahashi , and M. Nagino , “Portal Vein Embolization Before Extended Hepatectomy for Biliary Cancer: Current Technique and Review of 494 Consecutive Embolizations,” Digestive Surgery 29 (2012): 23–29. 22441616 10.1159/000335718

[6] K. P. Van Lienden , J. W. Van Den Esschert , W. De Graaf , et al., “Portal Vein Embolization Before Liver Resection: A Systematic Review,” Cardiovascular and Interventional Radiology 36 (2013): 25–34. 22806245 10.1007/s00270-012-0440-y PMC3549243

[7] R. Higuchi and M. Yamamoto , “Indications for Portal Vein Embolization in Perihilar Cholangiocarcinoma,” Journal of Hepato‐Biliary‐Pancreatic Sciences 21 (2014): 542–549. 24520045 10.1002/jhbp.77

[8] J. Shindoh , C. W. Tzeng , T. A. Aloia , et al., “Safety and Efficacy of Portal Vein Embolization Before Planned Major or Extended Hepatectomy: An Institutional Experience of 358 Patients,” Journal of Gastrointestinal Surgery 18 (2014): 45–51. 24129824 10.1007/s11605-013-2369-0

[9] J. C. Mansour , T. A. Aloia , C. H. Crane , J. K. Heimbach , M. Nagino , and J. N. Vauthey , “Hilar Cholangiocarcinoma: Expert Consensus Statement,” HPB: The Official Journal of the International Hepato Pancreato Biliary Association 17 (2015): 691–699. 26172136 10.1111/hpb.12450 PMC4527854

[10] M. C. Giglio , A. Giakoustidis , A. Draz , et al., “Oncological Outcomes of Major Liver Resection Following Portal Vein Embolization: A Systematic Review and Meta‐Analysis,” Annals of Surgical Oncology 23 (2016): 3709–3717. 27272106 10.1245/s10434-016-5264-6

[11] N. Ironside , R. Bell , A. Bartlett , J. McCall , J. Powell , and S. Pandanaboyana , “Systematic Review of Perioperative and Survival Outcomes of Liver Resections With and Without Preoperative Portal Vein Embolization for Colorectal Metastases,” HPB: The Official Journal of the International Hepato Pancreato Biliary Association 19 (2017): 559–566. 28438427 10.1016/j.hpb.2017.03.003

[12] G. K. Glantzounis , E. Tokidis , S. P. Basourakos , E. E. Ntzani , G. D. Lianos , and G. Pentheroudakis , “The Role of Portal Vein Embolization in the Surgical Management of Primary Hepatobiliary Cancers. A Systematic Review,” European Journal of Surgical Oncology 43 (2017): 32–41. 27283892 10.1016/j.ejso.2016.05.026

[13] G. Cassese , H. S. Han , B. Lee , et al., “Portal Vein Embolization Failure: Current Strategies and Future Perspectives to Improve Liver Hypertrophy Before Major Oncological Liver Resection,” World Journal of Gastrointestinal Oncology 14 (2022): 2088–2096. 36438704 10.4251/wjgo.v14.i11.2088 PMC9694272

[14] A. Takahashi , R. Yoshioka , M. Miyashita , et al., “Sequential Therapy of Portal Vein Embolization and Systemic Chemotherapy for Locally Advanced Perihilar Biliary Tract Cancer,” European Journal of Surgical Oncology 49 (2023): 150–155. 36089453 10.1016/j.ejso.2022.08.035

[15] A. Denys , P. Bize , N. Demartines , F. Deschamps , and T. De Baere , “Quality Improvement for Portal Vein Embolization,” Cardiovascular and Interventional Radiology 33 (2010): 452–456. 20130875 10.1007/s00270-009-9737-x PMC2868172

[16] F. Huisman , K. P. Cieslak , K. P. van Lienden , R. J. Bennink , and T. M. van Gulik , “Liver Related Complications in Unresectable Disease After Portal Vein Embolization,” Hepatobiliary Surgery and Nutrition 6 (2017): 379–386. 29312972 10.21037/hbsn.2017.02.03 PMC5756764

[17] R. Dhanasekaran , A. W. Hemming , I. Zendejas , et al., “Treatment Outcomes and Prognostic Factors of Intrahepatic Cholangiocarcinoma,” Oncology Reports 29 (2013): 1259–1267. 23426976 10.3892/or.2013.2290 PMC3621732

[18] S. K. Maithel , T. C. Gamblin , I. Kamel , C. P. Corona‐Villalobos , M. Thomas , and T. M. Pawlik , “Multidisciplinary Approaches to Intrahepatic Cholangiocarcinoma,” Cancer 119 (2013): 3929–3942. 23963845 10.1002/cncr.28312

[19] A. M. van Keulen , S. Franssen , L. G. van der Geest , et al., “Nationwide Treatment and Outcomes of Perihilar Cholangiocarcinoma,” Liver International 41 (2021): 1945–1953. 33641214 10.1111/liv.14856 PMC8359996

[20] T. Ioka , M. Kanai , S. Kobayashi , et al., “Randomized Phase III Study of Gemcitabine, Cisplatin Plus S‐1 Versus Gemcitabine, Cisplatin for Advanced Biliary Tract Cancer (KHBO1401‐MITSUBA),” Journal of Hepato‐Biliary‐Pancreatic Sciences 30 (2023): 102–110. 35900311 10.1002/jhbp.1219 PMC10086809

[21] L. T. Hoekstra , K. P. van Lienden , A. Doets , O. R. Busch , D. J. Gouma , and T. M. van Gulik , “Tumor Progression After Preoperative Portal Vein Embolization,” Annals of Surgery 256 (2012): 812–818. 23095626 10.1097/SLA.0b013e3182733f09

[22] Y. K. Yeom and J. H. Shin , “Complications of Portal Vein Embolization: Evaluation on Cross‐Sectional Imaging,” Korean Journal of Radiology 16 (2015): 1079–1085. 26357502 10.3348/kjr.2015.16.5.1079 PMC4559779

[23] A. Fukutomi , J. Furuse , T. Okusaka , et al., “Effect of Biliary Drainage on Chemotherapy in Patients With Biliary Tract Cancer: An Exploratory Analysis of the BT22 Study,” HPB: The Official Journal of the International Hepato Pancreato Biliary Association 14 (2012): 221–227. 22404259 10.1111/j.1477-2574.2011.00431.x PMC3371207

[24] J. Niemelä , R. Kallio , P. Ohtonen , J. Saarnio , and H. Syrjälä , “Impact of Cholangitis on Survival of Patients With Malignant Biliary Obstruction Treated With Percutaneous Transhepatic Biliary Drainage,” BMC Gastroenterology 23 (2023): 91. 36973653 10.1186/s12876-023-02704-8 PMC10041795

[25] M. Ueno , S. Shirakawa , J. Tokumaru , et al., “Real‐World Evidence of Systemic Treatment Practices for Biliary Tract Cancer in Japan: Results of a Database Study,” Journal of Hepato‐Biliary‐Pancreatic Sciences 31 (2024): 468–480. 38953871 10.1002/jhbp.1418 PMC11503459

