# Supplementary material for: Outcomes of Patients With Unresectable Cholangiocarcinoma After Portal Vein Embolization: A Propensity Score‐Matched Analysis
Source: J Hepatobiliary Pancreat Sci. 2025 Aug 1;32(11):819–28. doi: 10.1002/jhbp.12192 (PMC12648373; doi:10.1002/jhbp.12192)
Supplement: Supplementary file 1 — Figure S1. Comparison of survival and biliary drainage frequency between O&C and non‐laparotomy patients in the PVE‐unresectable group. [file JHBP-32-819-s001.docx]

**Supplementary Figure legend**

**Supplementary Figure S1. Comparison of Survival and Biliary Drainage Frequency Between O&C and Non-laparotomy Patients in the PVE-unresectable Group**

(A) Kaplan–Meier survival curves comparing patients in the PVE-unresectable group who underwent open-and-close laparotomy (O&C, n = 23) versus those who did not undergo surgical exploration (Non-laparotomy, n = 33). No statistically significant difference in overall survival was observed between the two groups (log-rank *p* = 0.62). Shaded areas indicate 95% confidence intervals, and the number at risk is shown below the time axis.

(B) Monthly biliary drainage (BD) frequency among patients in the PVE-unresectable group, stratified by surgical exploration status. No statistically significant difference was observed between the non-laparotomy group (n = 33) and the open-and-close group (n = 23) (Wilcoxon rank sum test, *p* = 0.4338). Boxplots indicate the median and interquartile ranges, and individual data points are shown.
