# Supplementary figures and images for: Outcomes of Patients With Unresectable Cholangiocarcinoma After Portal Vein Embolization: A Propensity Score‐Matched Analysis
Source: J Hepatobiliary Pancreat Sci. 2025 Aug 1;32(11):819–28. doi: 10.1002/jhbp.12192 (PMC12648373; doi:10.1002/jhbp.12192)

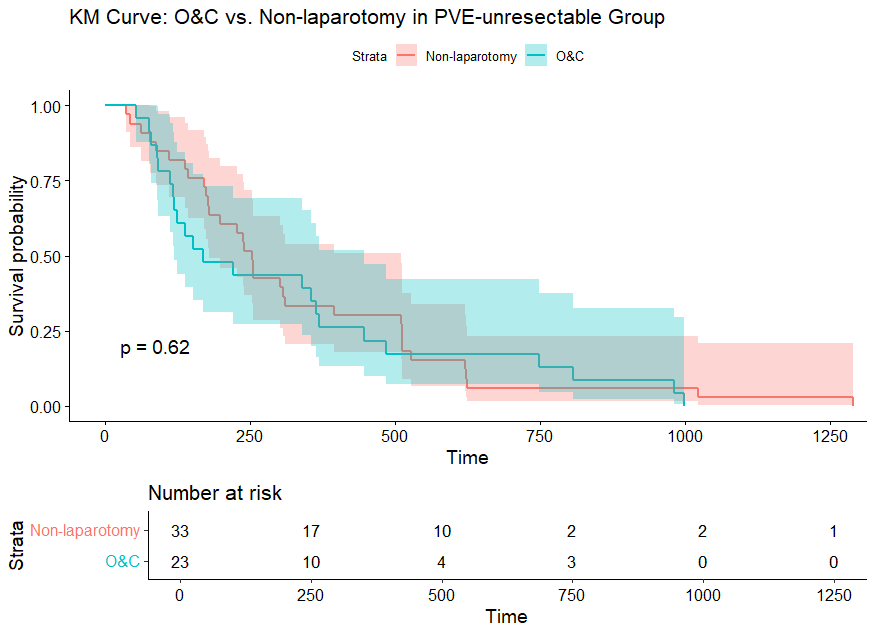

Supplement: Supplementary file 2 — Data S1. [file JHBP-32-819-s002.tiff]

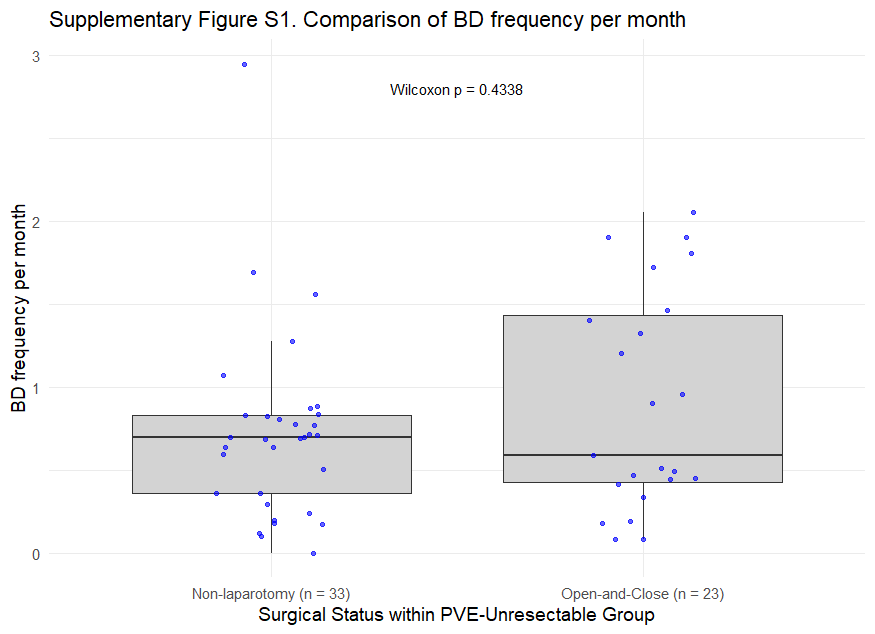

Supplement: Supplementary file 3 — Data S2. [file JHBP-32-819-s003.tiff]
