# Supplementary material for: Outcomes of Patients With Unresectable Cholangiocarcinoma After Portal Vein Embolization: A Propensity Score‐Matched Analysis
Source: J Hepatobiliary Pancreat Sci. 2025 Aug 1;32(11):819–28. doi: 10.1002/jhbp.12192 (PMC12648373; doi:10.1002/jhbp.12192)
Supplement: Supplementary file 4 — Table S1. Indications for first biliary drainage after portal vein embolization (PVE) in the PVE‐unresectable group. Table S2. Reasons for non‐resection after portal vein embolization. [file JHBP-32-819-s004.docx]

**Supplementary Table S1.** **Indications for First Biliary Drainage After Portal Vein Embolization (PVE) in the PVE-Unresectable Group**

| Indication | Number of Patients (%) |
| --- | --- |
| Cholangitis, liver abscess or worsening jaundice | 39 (69.6%) |
| Photodynamic therapy | 1 (1.8%) |
| For biopsy | 1 (1.8%) |
| Change plastic stent to metal stent through endoscopy | 5 (8.9%) |
| Stent insertion through PTBD | 2 (3.6) |
| No biliary drainage performed after PVE | 8 (14.3%) |

PVE, portal vein embolization; PTBD, percutaneous transhepatic biliary drainage

**Supplementary Table S1.** Indications for the first biliary drainage procedure performed after portal vein embolization in the PVE-unresectable group. The majority of procedures were therapeutic in nature, most commonly for cholangitis, liver abscess or worsening jaundice.

**Supplementary Table S2. Reasons for Non-Resection After Portal Vein Embolization**

| **Reason** | **Number of Patients (%)** |
| --- | --- |
| Sepsis due to cholangitis and/or general weakness | 16 (28.6%) |
| Insufficient remnant liver volume | 9 (16.1%) |
| Peritoneal seeding | 9 (16.1%) |
| Major vessel invasion | 7 (12.5%) |
| Hilar and/or duodenal invasion | 6 (10.7%) |
| Hepatic metastasis | 4 (7.1%) |
| Liver cirrhosis | 2 (3.6%) |
| Paraaortic lymph node metastasis | 1 (1.8%) |
| Pancreas invasion | 1 (1.8%) |
| Other medical condition (severe left internal carotid artery stenosis) | 1 (1.8%) |
